# Biases from different DNA extraction methods in intestine microbiome research based on 16S rDNA sequencing: a case in the koi carp, *Cyprinus carpio* var. *Koi*

**DOI:** 10.1002/mbo3.626

**Published:** 2018-04-17

**Authors:** Zhuoran Han, Jingfeng Sun, Aijun Lv, Anli Wang

**Affiliations:** ^1^ Key Laboratory of Ecology and Environment Science of Higher Education Institutes Guangdong Provincial Key Laboratory for Healthy and Safe Aquaculture College of Life Science South China Normal University Guangzhou China; ^2^ Tianjin Key Lab of Aqua‐ecology and Aquaculture Fisheries College Tianjin Agricultural University Tianjin China

**Keywords:** 16S ribosomal DNA, high‐throughput sequencing, intestinal microflora, Koi carp

## Abstract

This study examined the technical bias associated with different DNA extraction methods used in microbiome research. Three methods were used to extract genomic DNA from the same intestinal microbiota sample that was taken from the koi carp *Cyprinus carpio* var. *koi*, after which their microbial diversity and community structure were investigated on the basis of a 16S rDNA high‐throughput sequencing analysis. Biased results were observed in relation to the number of reads, alpha diversity indexes and taxonomic composition among the three DNA extraction protocols. A total of 1,381 OTUs from the intestinal bacteria were obtained, with 852, 759, and 698 OTUs acquired, using the Lysozyme and Ultrasonic Lysis method, Zirmil‐beating Cell Disruption method, and a QIAamp Fast DNA Stool Mini Kit, respectively. Additionally, 336 OTUs were commonly acquired, using the three methods. The results showed that the alpha diversity indexes (Rarefaction, Shannon, and Chao1) of the community that were determined using the Lysozyme and Ultrasonic Lysis method were higher than those obtained with the Zirmil‐beating Cell Disruption method, while the Zirmil method results were higher than those measured, using the QIAamp Fast DNA Stool Mini Kit. Moreover, all the major phyla (ratio>1%) could be identified with all three DNA extraction methods, but the phyla present at a lower abundance (ratio <1%) could not. Similar findings were observed at the genus level. Taken together, these findings indicated that the bias observed in the results about the community structure occurred primarily in OTUs with a lower abundance. The results of this study demonstrate that possible bias exists in community analyses, and researchers should therefore be conservative when drawing conclusions about community structures based on the currently available DNA extraction methods.

## 1 INTRODUCTION

Asian‐origin koi carp (*Cyprinus carpio* var. *Koi*) are currently listed among the most important ornamental species because they can be reared in all the countries in the world. Their broad diversity of colors and color patterns are major factors contributing to their attractive market value (David et al., 2004). However, various infectious diseases in koi carp have emerged with the rapid development of industrial culture in recent years, resulting in great economic losses (Kumar et al., 2015; Pokorova et al., 2007).

Gut microbiota play important roles in fish health and physiology (Ganguly & Prasad, 2011). Studies have shown that gut microbiota are associated with many key functions of the host, such as resistance to infectious diseases and the decomposition of nutrients, and they provide the host with physiologically active materials including enzymes, amino acids and vitamins (Sugita, Kawasahi, & Deguchi, 1997). Accordingly, altered microbiota in the intestine can lead to changes in host immune functions as well as an increased risk of disease (Brown, DeCoffe, Molcan, & Gibson, 2012; Morgan et al., 2012). Over the last decade, an increasing number of studies have focused on the gut microbiota of fish (Narrowe et al., 2015; Xia et al., 2014). In early studies, conventional culture‐dependent techniques were used; however, only a small percentage of the resulting bacterial flora was identified (Kathiravan et al., 2015). Recently, new technologies based on meta‐genomics/high‐throughput sequencing have been developed and successfully applied to analyzing the complex bacterial ecosystem of the gut. These new analytical approaches usually involve DNA extraction from stool samples or biopsies and the amplification of 16S ribosomal DNA (rDNA) followed by high‐throughput sequencing. Increasing evidence shows that 16S rDNA sequencing approaches can be used to identify bacteria rapidly because they can overcome the limitations of culture‐based bacterial detection methods.

The extraction of DNA from intestinal fecal samples is a key step in molecular biological analyses. Several protocols for extracting DNA from fish intestinal microflora have been described, including physical and chemical methods. Generally, common physical disruption methods have been employed, including freezing‐thawing (Silva, Bernardi, Schaker, Menegotto, & Valente, 2012), sonication (Yang, Xiao, Zeng, Liu, & Deng, 2006) and bead beating (Carrigg, Rice, Kavanagh, Collins, & O'Flaherty, 2008). In addition, a variety of chemical lysis approaches has been used to obtain higher purity DNA samples, including cetyltrimethylammonium bromide (CTAB) (Chapela et al., 2007). However, different DNA extraction protocols can lead to biases with respect to the microbial diversity, community structure, proportions and number of reads and numbers of OTUs obtained based on the 16S rDNA high‐throughput sequencing, subsequently influencing estimations of the microbial diversity and the taxonomic composition in the intestinal mucosa and intestinal content. Moreover, because there is no “gold standard” method for DNA extraction, it is difficult to determine the “true” diversity of the bacterial community. Some have suggested combining several extraction methods, if possible, to recover some of the loss in observable diversity that occurs when only one DNA extraction is used (Kashinskaya, Andree, Simonov, & Solovyev, 2016; Wen, He, Xue, Liang, & Dong, 2016).

This study examined the bias in results that were obtained using different extraction methods during microbiome research based on 16S rDNA high‐throughput sequencing analyses. Three methods were used to extract the genomic DNA from the same sample of intestinal microbiota from the koi carp, *Cyprinus carpio* var. *koi*. Specifically, a protocol was modified from the lysozyme method developed by our laboratory and named the Combination of Lysozyme and Ultrasonic Lysis method (CLU); the Zirmil‐beating Cell Disruption method (ZBC) referring to the research of Zoetendal et al. (2006) and a QIAamp Fast DNA Stool Mini Kit (QIA, Qiagen, Hilden, Germany), a common commercial kit, were also used.

## 2 MATERIALS AND METHODS

### 2.1 Sample preparation for DNA isolation

Koi carp were provided by the Gongwang koi fish‐breeding center in Tianjin, China. The fish were transported to Tianjin Agricultural University, where they were maintained under optimal rearing conditions for 1 week in 20°C water. Aeration was provided to maintain optimal dissolved oxygen levels and the fish were fed commercial pellets twice daily. Genomic DNA was extracted from the intestinal contents and mucosa of adult koi carp that were 30–35 cm long and 380–410 g. In brief, the fish were euthanized with an overdose of MS‐222 (Sigma‐Aldrich, St Louis, MO, USA), after which their exteriors were wiped clean with 70% ethanol, their abdomens were opened at the ventral midline and the whole intestines were aseptically removed from the abdominal cavity. All the experimental procedures performed on these koi carp were approved by the Animal Care Committee of Tianjin Agricultural University, and the methods were performed in accordance with the approved guidelines and regulations.

The gut samples were used directly after their removal from the fish. The intestinal contents and mucosa of three fish were collected into a 50‐ml centrifuge tube and homogenized in 15 ml of sterile phosphate‐buffered solution (PBS, 0.01 mol/L, pH 7.2; Dingguo Changsheng, Beijing, China) by vortexing (IKA, Germany) three times at 158 g for 20 s each. The samples were then centrifuged at 110 g for 5 min at 4°C, after which the supernatant was dispensed into a new sterile 50‐ml centrifuge tube. The supernatant was subsequently centrifuged at 2,739 g for 5 min. The bacterial precipitation was then resuspended in 3 ml of PBS. The bacterial suspension of mucosa and intestinal content from the three koi carp was divided into triplicate samples. One milliliter of bacterial suspension was used for the CLU method, one for the ZBC method, and another one for the QIA method.

### 2.2 DNA extraction

#### 2.2.1 CLU method

The bacterial suspension (1 ml) was dispensed into a 2‐ml microtube, and then it was disrupted using an Ultrasonic Cell Disruption System (Ningbo Scientz Biotechnology, Ningbo, China) 50 times for 2 s each with an interval of 5 s between each disruption. Next, the samples were centrifuged at 15,777 g for 5 min at 4°C, after which the upper aqueous layer was discarded. Each sample was then incubated for 30 min at 60°C in 750 μl of TE (10 mmol/L Tris‐HCl, 1 mmol/L EDTA, pH 8.0) and 50 μl of lysozyme (20 mg/ml; Sangon Biotech, Shanghai, China). Subsequently, 10 μl of RNase A (20 μg/ml; Sangon Biotech) was added to the centrifuge tube, after which the suspension was incubated for 30 min at 30°C. The tube was then incubated for 60 min at 65°C with inversion every 20 min after adding 100 μl of 10% SDS (0.1 g/ml, pH 7.4; Sigma Aldrich) and 30 μl of Proteinase K (20 mg/ml; Sangon Biotech). Thereafter, an equal volume of phenol: chloroform: isoamyl alcohol (25: 24: 1) was added and mixed by inversion. The samples were then centrifuged at 15,777 g for 2 min, after which the supernatant was collected in a new sterile 2‐ml centrifuge tube. An equal volume of chloroform: isoamyl alcohol (24: 1) was then added to the tube, after which the suspension was mixed gently and centrifuged at 15,777 g for 2 min. The upper aqueous layer was subsequently transferred to another 2‐ml sterile centrifuge tube, and the DNA was then precipitated using a 1/10 volume of NaAc (3 mol/L, pH 5.2) and 2 volumes of ice‐cold (−20°C) 95% ethanol, followed by centrifugation at 15,777 g for 5 min at 4°C. Finally, the DNA pellet was washed twice with 1 ml of 70% ethanol before it was air‐dried and finally resuspended in 100 μl of TE buffer that had been preheated to 50°C.

#### 2.2.2 ZBC method

DNA was extracted from 1 ml of bacterial suspension according to the modified ZBC method (Zoetendal et al., 2006). In brief, the bacterial suspension was transferred to a 2‐ml Lysing Matrix A tube (MP Biomedicals, Santa Ana, CA, USA), after which 150 μl of buffer‐saturated phenol was added to the tube. The sample was then oscillated at 4 m/s for 2 min, using a FastPrep^®^‐24 Instrument (MP Biomedicals), then cooled on ice for 30 s and purified with 150 μl chloroform: isoamyl alcohol (24: 1), and after that it was centrifuged at 15,777 g for 2 min at 4°C. At that stage, an equal volume of phenol: chloroform: isoamyl alcohol (25: 24: 1) was added and mixed in by inversion. Next, the sample was centrifuged at 15,777 g for 2 min and the supernatant was transferred to a new 2‐ml sterile centrifuge tube. This step was repeated until the interface of the two layers was clean, after which an equal volume of chloroform: isoamyl alcohol (24: 1) was added to the tube. The sample was then mixed gently and centrifuged at 15,777 for 2 min, after which the supernatant was transferred into a new 2‐ml centrifuge tube. Next, the DNA was precipitated with 1/10 volume of 3 mol/L NaAc (pH 5.2) and 2 volumes of cold 95% ethanol (−20°C) and stored at −20°C for 30 min. The samples were then centrifuged at 15,777 g for 10 min, and the supernatant was discarded. The DNA was washed with 1 ml of cold (−20°C) 70% ethanol and centrifuged at 15,777 g for 5 min at 4°C. Finally, the DNA pellet was dried by placing the tube upside down on tissue paper for 15 min, after which the dried DNA was rehydrated in 100 μl of TE buffer.

#### 2.2.3 QIA method

One‐milliliter bacterial suspensions were centrifuged at 2,739 g for 5 min, after which the bacterial precipitation was resuspended with 220 μl of PBS. Next, DNA was extracted from 220 μl of bacterial suspension using a QIAamp Fast DNA Stool Mini Kit (Qiagen, Hilden, Germany) according to the manufacturer's instructions.

### 2.3 High‐throughput 16S rDNA Illumina MiSeq sequencing

To analyze the microbial populations of the extracted DNA samples, the variable V3‐V4 region of the 16S rDNA was amplified. To this end, a polymerase chain reaction (PCR) was conducted using the bacterial universal primers 341F (5′‐CCCTACACGACGCTCTTCCGATCTGCCTACGGGNGGCWGCAG‐3′) and 805R (5′‐GACTGGAGTTCCTTGGCACCCGAGAATTCCAGACTACHVGGGTATCTAATCC‐3′) (Li et al., 2016). Barcodes unique to each sample were incorporated before the forward primers, which allowed for the identification of each sample in a mixture for an Illumina sequencing run. Each reaction was performed in a 50‐μl volume containing 20 ng of bacterial DNA, 5 μl of 10 × PCR buffer, 0.5 μl of dNTP (10 mmol/L), 0.5 μl of Bar‐PCR primer F (50 μmol/L), 0.5 μl of Primer R (50 μmol/L), and 0.5 μl of Platinum Taq (5 U/μl), with ddH_2_O added to 50 μl. The samples were subsequently amplified, using a T100^™^ Thermal Cycler (BioRad, Hercules, CA, USA) under the following conditions: initial denaturation at 94°C for 3 min followed by 5 cycles of 94°C for 30 s, 45°C for 20 s, and 65°C for 30 s, then 20 cycles of 90°C for 30 s, 55°C for 20 s, and 72°C for 30 s, and then a final elongation at 72°C for 5 min. The PCR products were separated by electrophoresis in 2% agarose gels, purified with a SanPrep Column DNA Gel Extraction Kit (Sangon Biotech) and quantified using Qubit 2.0 (Thermo Scientific, DE, USA). Finally, the PCR products were sequenced and analyzed on an Illumina MiSeq platform according to the manufacturer's recommendations.

### 2.4 Data analysis

Following sequencing with the Illumina MiSeq, the sequencing reads were assigned to each sample according to their unique barcode. Pairs of reads from the original DNA fragments were first merged, using FLASH (Magoč & Salzberg, 2011). A quality control procedure was used, including trimming the barcodes and primers and filtering low‐quality reads by PRINSEQ (Schmieder & Edwards, 2011). The sequences that passed the above procedure were then denoised to correct for potential sequencing errors, and reads were discarded if they were identified by UCHIME as putative chimeras (Edgar, Haas, Clemente, Quince, & Knight, 2011). Finally, the filtered sequences were obtained. These sequences were classified into the same operational taxonomic units (OTUs) at an identity threshold of 97% similarity, using the Ribosomal Database Project (RDP) classifier (Wang, Garrity, Tiedje, & Cole, 2007). The Rarefaction, Shannon and Chao1 indexes were included in the alpha diversity analysis, using Mothur (Schloss et al., 2009). Weighted UniFrac metric distances were calculated to determine the beta diversity index, and the sample tree was used to examine the relationship of the community structures of the microbiota from different samples.

## 3 RESULTS

### 3.1 OTUs and alpha diversity analysis

**Figure 1:**
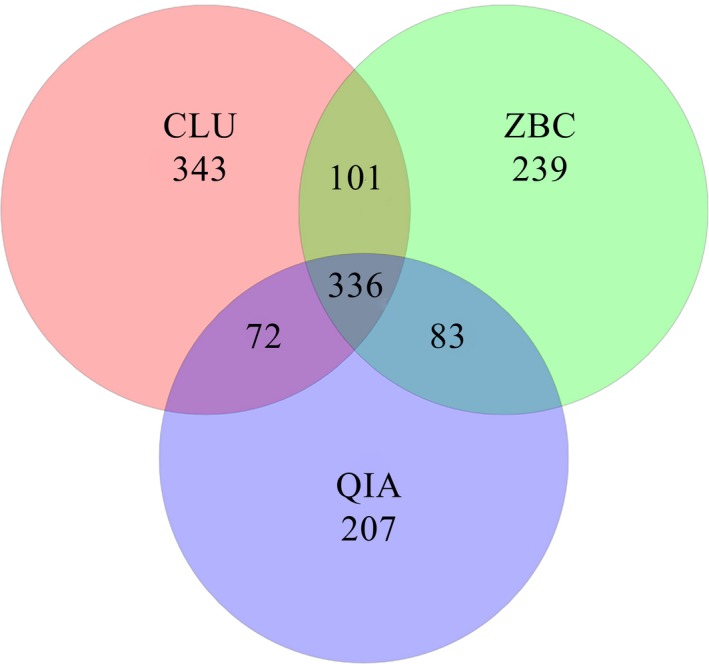
Venn diagram showing the OTUs obtained using the three DNA extraction methods. The three methods shared 336 OTUs among the communities identified in the genomic DNA samples. Overall, 852, 759, and 698 OTUs were obtained by CLU, ZBC and QIA methods, respectively

After applying quality control measures and filtering the chimera, the numbers of filtered reads (Filtered‐num) were 29,618, 41,379, and 48,586 for the DNA samples extracted, using the CLU, ZBC, and QIA methods, respectively (Table 1). These reads, which had a mean length of 415.7 bp, were assigned to 1,381 OTUs of intestinal bacteria based on a 97% similarity cut‐off (Figure 1). The numbers of OTUs were 852, 759, and 698 for samples extracted, using the CLU, ZBC, and QIA methods, respectively (Table 1). Moreover, samples extracted using the three methods shared 336 OTUs, accounting for 39.44%, 44.27%, and 48.14% of the respective OTU numbers for the CLU, ZBC, and QIA methods.

**Table 1:** Alpha diversity index, number of OTUs and filtered reads from DNA samples extracted with the CLU, ZBC or QIA methods

Sample	Filtered number	Mean length	OTUs	Shannon index	Chao1 index
CLU	29,618	421.2	852	3.41	1,609
ZBC	41,379	412.9	759	2.61	1,458
QIA	48,586	414.9	698	2.57	1,320

**Figure 2:**
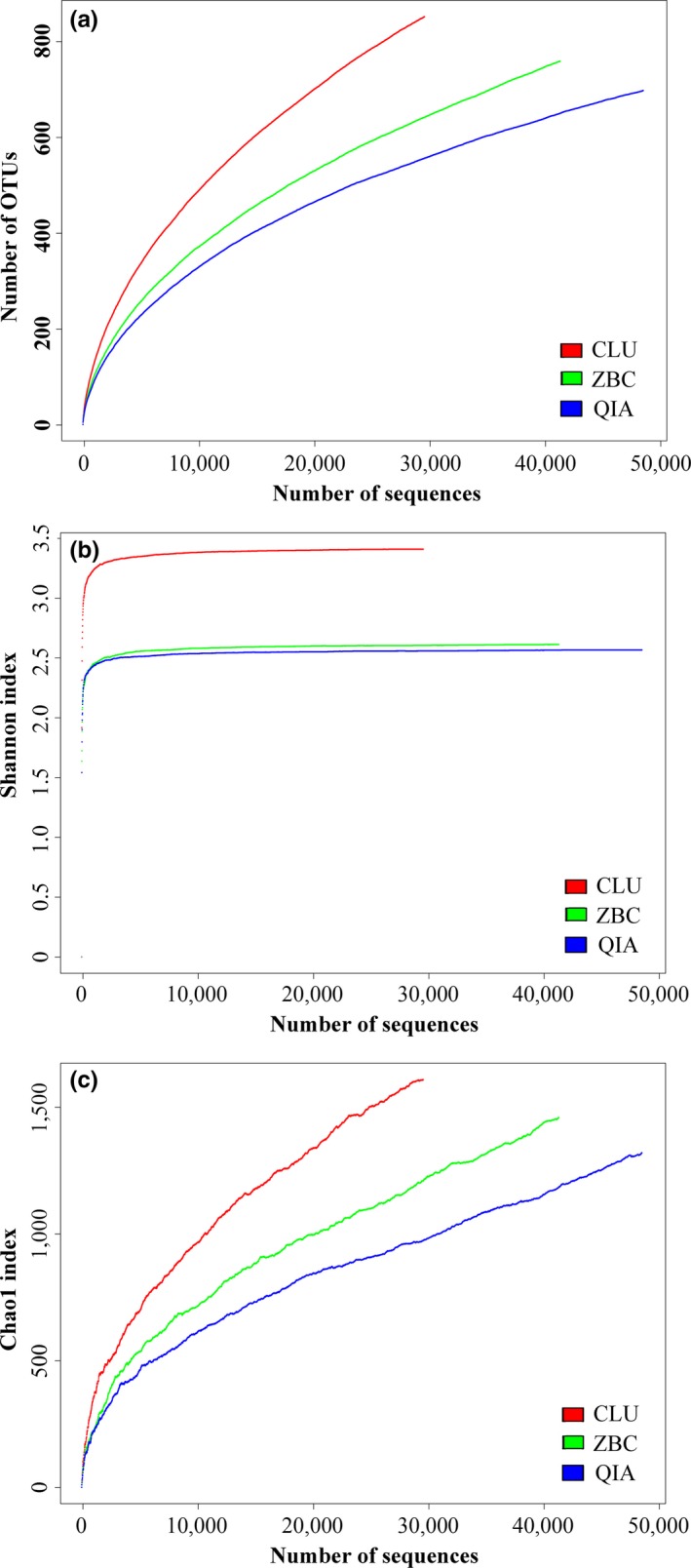
Alpha diversity associated with the genomic DNA samples extracted using the CLU, ZBC and QIA methods. **(**a): Rarefaction curves; (b): Shannon's diversity index; and (c): Chao1 index

The Rarefaction, Shannon's and Chao1 alpha diversity indexes were also calculated (Figure 2, Table 1). The ZBC and QIA methods showed similar trends in the rarefaction curves. However, the CLU method had a higher slope for the rarefaction curve than the other two methods (Figure 2a). The Shannon indexes were 3.41, 2.61, and 2.57 for the CLU, ZBC, and QIA methods, respectively (Figure 2b), while the Chao1 index values were 1,609, 1,458, and 1,320 for these methods (Figure 2c).

### 3.2 Bacterial community

**Figure 3:**
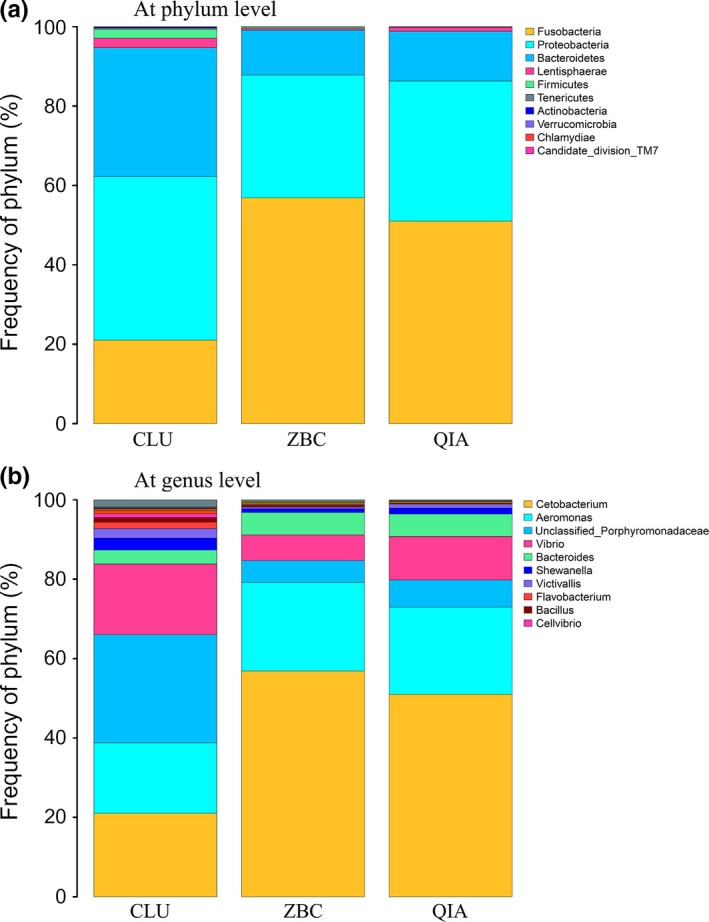
Predominant microbiomes identified from the DNA samples extracted using all three methods. (a): phylum level; and (b): genus level

The 10 OTUs with the highest abundance at the phylum level that were obtained, using the three extraction methods were identified (Figure 3a) and shown to belong to Fusobacteria, Proteobacteria, Bacteroidetes, Lentisphaerae, Firmicutes, Tenericutes, Actinobacteria, Verrucomicrobia, Chlamydiae, and Candidate_division_TM7. All ten OTUs from the microorganisms were identified in the DNA sample extracted, using the CLU method, while all except Candidate_division_TM7 were identified by ZBC method, and all except Candidate_division_TM7 and Chlamydiae were identified by QIA method. In addition, more than 94% of the sequences in all the samples were found to belong to the three most populated bacterial phyla, Fusobacteria, Proteobacteria, and Bacteroidetes. The ZBC method showed 98.80% for the three bacterial phyla, while those of the QIA and CLU methods were 98.80% and 94.71%, respectively (Table 2).

**Table 2:** Ten OTUs from microorganisms with the highest abundance at the phylum level were identified on the basis of DNA samples extracted using all three methods

Name	CLU reads	CLU ratio (%)	ZBC reads	ZBC ratio (%)	QIA reads	QIA ratio (%)
Fusobacteria	4,888	21.04	18,469	56.89	19,817	51.00
Proteobacteria	9,591	41.28	10,048	30.95	13,722	35.30
Bacteroidetes	7,525	32.39	3,661	11.28	4,855	12.50
Lentisphaerae	562	2.42	135	0.42	387	1
Firmicutes	507	2.18	112	0.34	39	0.1
Tenericutes	78	0.34	16	0.05	25	0.06
Actinobacteria	46	0.2	10	0.03	1	0
Verrucomicrobia	27	0.12	7	0.02	6	0.02
Chlamydiae	5	0.02	2	0.01	0	0
Candidate_division_TM7	2	0.01	0	0	0	0

The CLU, ZBC or QIA reads represent the read numbers of identified microorganisms in DNA extracted using the CLU, ZBC, or QIA methods, respectively. The CLU, ZBC or QIA ratio represent the ratio of sequencing reads from microorganisms in the DNA extracted using the CLU, ZBC, QIA methods, respectively.

At the genus level (Figure 3b), the 10 OTUs of microorganisms with the highest abundance based on the three extraction methods were *Cetobacterium*,* Aeromonas*, unclassified_Porphyromonadaceae, *Vibrio*,* Bacteroides*,* Shewanella*,* Victivallis*,* Flavobacterium*,* Bacillus*, and *Cellvibrio*. Additionally, *Cetobacterium*,* Aeromonas*, unclassified_Porphyromonadaceae, *Vibrio*, and *Bacteroides* were identified as the five most abundant bacterial taxa, and they accounted for 87.33%, 96.81%, and 96.40% of those identified by the CLU, ZBC, and QIA methods, respectively (Table 3).

**Table 3:** Ten OTUs of microorganisms with the highest abundance at the genus level were identified on the basis of the DNA samples extracted using all three methods

Name	CLU reads	CLU ratio (%)	ZBC reads	ZBC ratio (%)	QIA reads	QIA ratio (%)
*Cetobacterium*	4,881	21.01	18,467	56.88	19,810	50.99
*Aeromonas*	4,134	17.79	7,256	22.35	8,532	21.96
unclassified_Porphyromonadaceae	6,327	27.23	1,780	5.48	2,660	6.85
*Vibrio*	4,137	17.81	2,097	6.46	4,262	10.97
*Bacteroides*	811	3.49	1,832	5.64	2,187	5.63
*Shewanella*	701	3.02	305	0.94	589	1.52
*Victivallis*	562	2.42	135	0.42	387	1
*Flavobacterium*	369	1.59	39	0.12	5	0.01
*Bacillus*	4	0.02	0	0	0	0
*Cellvibrio*	227	0.98	53	0.16	43	0.11

The CLU, ZBC or QIA reads represent the read numbers of the microorganisms identified in DNA extracted using the CLU, ZBC or QIA methods, respectively. The CLU, ZBC or QIA ratio represent the sequencing read ratios of microorganisms identified in the DNA samples using the CLU, ZBC or QIA methods, respectively.

At the species level (Table 4), 17 OTUs were detected. The three methods shared four species of microorganisms, *Aeromonas veronii*,* Chitinilyticum aquatile*,* Deefgea chitinilytica*, and *Vibrio cholera*. Six species (*Anaerorhabdus furcosa*,* Bacillus aryabhattai*,* Bacillus horikoshii*,* Cellulomonas gelida*,* Flavobacterium tilapiae*, and *Vibrio lentus*) were identified only in the DNA sample extracted with the CLU method, while four species (*Aeromonas sharmana*,* Aeromonas hydrophila*,* Pseudomonas mosselii*, and *Rhodococcus zopfii*) were identified only in the results obtained, using the QIA method. *Aeromonas caviae* was only one that was detected in the sample acquired by ZBC method.

**Table 4:** Seventeen OTUs from microorganisms at the species level were identified based on DNA samples extracted using all three methods

Name	CLU reads	CLU ratio (%)	ZBC reads	ZBC ratio (%)	QIA reads	QIA ratio (%)
*Aeromonas sharmana*	0	0	0	0	1	0
*Aeromonas hydrophila*	0	0	0	0	4	0.01
*Aeromonas veronii*	13	0.06	24	0.07	48	0.12
*Aeromonas caviae*	0	0	1	0	0	0
*Anaerorhabdus furcosa*	1	0	0	0	0	0
*Bacillus aryabhattai*	1	0	0	0	0	0
*Bacillus horikoshii*	2	0.01	0	0	0	0
*Cellulomonas gelida*	1	0	0	0	0	0
*Chitinilyticum aquatile*	36	0.15	32	0.1	6	0.02
*Deefgea chitinilytica*	2	0.01	1	0	1	0
*Flavobacterium tilapiae*	1	0	0	0	0	0
*Pseudomonas mosselii*	0	0	0	0	1	0
*Pasteurella pneumotropica*	9	0.04	0	0	2	0.01
*Rhodococcus zopfii*	0	0	0	0	1	0
*Vibrio cholerae*	4,026	17.33	1587	4.89	3,669	9.44
*Vibrio vulnificus*	4	0.02	3	0.01	0	0
*Vibrio lentus*	47	0.2	0	0	0	0

The CLU, ZBC or QIA reads represent the read number of microorganisms identified in DNA extracted using the CLU, ZBC or QIA methods, respectively. The CLU, ZBC or QIA ratio represent the ratio of sequencing reads from microorganisms identified in DNA extracted using the CLU, ZBC or QIA methods, respectively.

### 3.3 Beta‐diversity analysis

**Figure 4:**
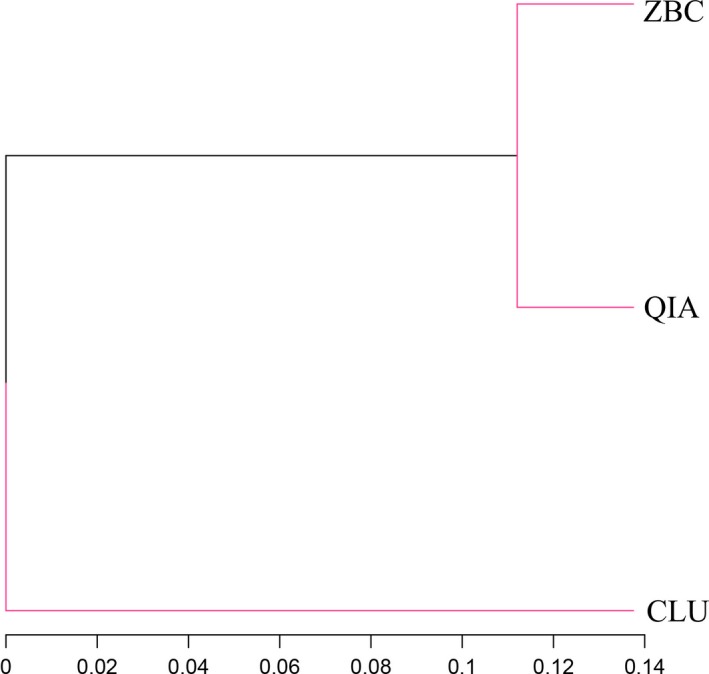
Distance‐based weighted UniFrac analysis associated with the three genomic DNA extraction methods. The microbial community structures and species richness of DNA samples extracted using the ZBC and QIA methods were more similar than those obtained using the CLU and ZBC methods or the CLU and QIA methods

The relationship among the community structures of the microbiota from different samples was examined using a sample tree based on the weighted UniFrac distance matrixes. The microbial community structures and species richness were more similar between samples extracted, using the ZBC and QIA methods than those obtained, using the CLU and ZBC methods or the CLU and QIA methods (Figure 4).

## 4 DISCUSSION

Gut microbiota can play important roles in nutrition and health, and they may be considered an integral component of the host (Artis, 2008; Ganguly & Prasad, 2011). Recently, molecular techniques have been used successfully to analyze the complex microbial community of the fish intestine (Gajardo et al., 2016; Xia et al., 2014). Most of these approaches focus on the use of 16S rRNA and its corresponding gene because 16S rRNA is present in every cell, and it has a low mutation and horizontal transfer rate. Therefore, it has been used as a phylogenetic marker for the detection, identification, and quantification of unculturable microbes from a variety of ecosystems, including animal GI tracts. The first and most important step in the 16S rRNA approach is the reliable isolation of nucleic acids from tract samples because the quality of all the subsequent procedures is dependent on this step. Although a great number of DNA extraction methods have been used to evaluate fish gut microbiota, a previous study demonstrated that the extraction step introduces bias into the observed community structure and the microbial diversity of fish intestines (Kashinskaya et al., 2016).

In the present study, three DNA extraction methods were used to extract the genomic DNA of intestinal microbiota from the koi carp *C. carpio* var. *Koi*, and their microbial diversity and community structure were detected using a 16S rDNA high‐throughput sequencing analysis. We compared three different methods of extracting microbial DNA from the same sample by analyzing the given taxonomic composition and microbial diversity. Biases in the results obtained among the three DNA extraction protocols were observed in relation to the number of reads, alpha diversity indexes and taxonomic composition. Specifically, the results showed that the alpha diversity indexes (Rarefaction, Shannon, and Chao1) of the community obtained, using the CLU method were higher than those acquired by the ZBC method, while those of the ZBC method were higher than those obtained using the QIA method. The rarefaction curves associated with the three methods did not reached saturation. Although the sampling depth for the sequence analysis was the same for the DNA samples extracted with the three methods, the CLU method presented a more positive slope for the rarefaction curve than the ZBC and OIA methods. This finding is indicative that further sampling would reveal more species richness for the CLU method than for the ZBC and QIA methods. The results of the Chao1 index showed that the potential number of species was greater in the DNA sample obtained by the CLU method than with the ZBC and QIA methods, which was similar to the results of the rarefaction curves. In addition, less than 50% of the OTUs identified in the DNA samples were shared among samples acquired using different methods. These results were consistent with those of the previous studies (Kashinskaya et al., 2016; Wen et al., 2016), indicating that different protocols introduce a bias into the observed results relating to the microbial diversity and richness in fish intestines.

It is well known that different bacterial groups (gram‐negative, gram‐positive, etc.) demonstrate different degrees of resistance to the chemical agents applied during DNA extraction (von Wintzingerode, Gobel, & Stackebrandt, 1997). In some cases, the chemical agents cannot break the bacterial cell walls completely, preventing the DNA from being released into solution; whereas in other cases, the chemical agents might lyse the cells well but damage the DNA in the process. These factors could lead to the under‐ or overestimation of different bacterial groups in the microbial community.

During the CLU method, lysozyme and ultrasonic lysis were combined to extract the genomic DNA. It was reported that lysozyme had only modest DNA extraction efficiency for gram‐positive bacteria and a few gram‐negative bacteria (Yu, Sun, Li, & Sun, 2013). To compensate for the low DNA extraction efficiency of the lysozyme for most gram‐negative bacteria, a treatment step with proper ultrasonic disruption was used in the CLU method to breakdown gram‐negative bacteria cell walls and enable the very effective liberation of the DNA. Moreover, the collective actions of the hydrolytic enzymes Proteinase K and RNase A used in this method aided in the release and purification of the DNA. The ZBC method is based on mechanical disruption followed by DNA isolation, using Phenol: Chloroform: Isoamyl Alcohol extraction. The QIA method was adapted from a QIAamp Fast DNA Stool Mini Kit (Qiagen), which contains proprietary commercial ingredients whose constituents are not disclosed by the manufacturer. Nevertheless, the ZBC and QIA methods are less separated by the distance tree based on the weighted UniFrac analysis, resulting in the samples obtained by these two methods being more closely grouped.

Because of the multitude of different DNA extraction methods and analytical methods for investigating these samples, there is no universal method to evaluate the bacterial diversity of fish guts. However, Proteobacteria, Firmicutes, and Bacteroidetes were identified as the major phyla in the gut microbiota of the koi carp, which is similar to the results obtained for sea bream (*Sparus aurata*) (Kormas, Meziti, Mente, & Frentzos, 2014). Previous studies also revealed a core microbiome from the intestines of fish dominated by Proteobacteria, Firmicutes, Bacteroidetes, Actinobacteria, and Fusobacteria (Smriga, Sandin, & Azam, 2010; Sullam et al., 2012; Ye, Amberg, Chapman, Gaikowski, & Liu, 2014). In a study conducted by Kashinskaya et al. (2016), the intestinal microbiota of the Prussian carp, *Carassius gibelio*, was dominated by Proteobacteria and Firmicutes based on an analysis of DNA extracted using an AxyPrep Multisource Genomic DNA Miniprep Kit (Axygen Biosciences, Union City, California, USA), while Proteobacteria, Firmicutes, and Bacteroidetes were found to be dominant after the intestinal microbiota were analyzed according to the DNA extracted using a DNA‐sorb B kit (kit for DNA extraction, Central Research Institute of Epidemiology, Moscow, Russia). The results of our meta‐analysis have shown that these major groups of bacteria could all be identified, using the three DNA extraction methods employed herein. In addition, all major phyla (ratio >1%) could be identified through the analysis of DNA obtained by the three extraction methods used here, but the phyla that were present at a lower abundance (ratio <1%) could not. Similar findings were observed at the genus level. These results indicated that the bias in the observed community structures was primarily in OTUs present at a lower abundance.

Previous investigations have demonstrated that variations in the intestinal microbiota of different fish species depend on the diet, trophic level, intestinal microenvironment, age, geographical location, and environmental conditions (Bolnick et al., 2014; Clements, Angert, Montgomery, & Choat, 2014; Kashinskaya et al., 2016; Sullam et al., 2012; Uchii et al., 2006; Wong & Rawls, 2012; Ye et al., 2014). In the present study, the use of only one library construction method and a single primer set probably limited the ability to identify the observable diversity. In addition, the first centrifugation of 110 g used for the DNA isolation sample preparation aimed to discard the solid residue from the mucosa and intestinal contents. This step would unavoidably result in some loss from the bacterial sample and consequently some loss in the potential biodiversity. To determine the “true” diversity of the bacterial community, the development of a universal methodology that is applicable to more samples is needed. The results of the present study suggested that bias is present among DNA extraction methods; therefore, researchers should be conservative in drawing conclusions about community structures.

## CONFLICT OF INTEREST

None declared.
